# Systematic Review of Myopia Progression after Cessation of Optical Interventions for Myopia Control

**DOI:** 10.3390/jcm13010053

**Published:** 2023-12-21

**Authors:** Yu-Chieh Chiu, Ping-Chiao Tsai, Ssu-Hsien Lee, Jen-Hung Wang, Cheng-Jen Chiu

**Affiliations:** 1 School of Medicine, Tzu Chi University, Hualien 970, Taiwan; hl2200571@tzuchi.com.tw (Y.-C.C.); hl2200544@tzuchi.com.tw (P.-C.T.); hl2200543@tzuchi.com.tw (S.-H.L.); 2 Department of Medical Research, Buddhist Tzu Chi General Hospital, Hualien 970, Taiwan; paulwang@tzuchi.com.tw; 3 Department of Ophthalmology and Visual Science, Tzu Chi University, Hualien 970, Taiwan; 4 Department of Ophthalmology, Hualien Tzu Chi Hospital, the Buddhist Tzu Chi Medical Foundation, Hualien 970, Taiwan

**Keywords:** myopia, myopia control, optical interventions, bifocal contact lens, spectacles, orthokeratology, MiSight, rebound effect, discontinuation

## Abstract

Despite high discontinuation rates for myopia optical interventions, limited attention has been given to the potential rebound effects post-discontinuation. This systematic review aims to assess the extent of the rebound effects following the cessation of common clinical optical myopia-control interventions in children. A comprehensive search of PubMed, Embase, Cochrane CENTRAL, and ClinicalTrials.gov was conducted from inception to October 2023. The rebound effects, defined as changes in the axial length or spherical equivalent during and after treatment cessation, were categorized into four levels. These studies encompassed 703 participants and spanned from 2019 to 2023, with durations of treatment and cessation ranging from 6 months to 3.5 years and from 2 weeks to 5 years, respectively. This review, encompassing 14 studies, revealed a predominant strong rebound effect in orthokeratology (8 studies), a weak rebound effect in multifocal soft contact lenses (4 studies), and a variable rebound effect in peripheral-plus spectacle lenses (2 studies). Notably, with the increasing cessation duration, the rebound effects diminished, potentially linked to the reversal of choroidal thickening and the disappearance of peripheral myopic defocus. In conclusion, a temporal trend of rebound effects exists in all three myopia optical interventions, possibly contributing to their myopia control mechanisms.

## 1. Introduction

The increasing global prevalence of myopia [1,2,3,4], which is especially rampant in East and Southeast Asia [1], has raised concerns about myopia-related visual impairment [5,6,7]. Projections suggest that by 2050, half of the global population will be myopic [2], marking this trend as a significant health concern in recent decades. Myopia is typically diagnosed in young children and can progress rapidly, necessitating interventions during childhood. Consequently, various strategies for slowing the progression of myopia have been explored, including pharmacologic approaches, such as topical atropine and optical interventions like orthokeratology, multifocal soft contact lenses (MFSCLs), and peripheral-plus spectacle lenses (PPSLs) [8,9,10].

Orthokeratology involves wearing cornea-reshaping lenses overnight to induce myopic defocus to slow axial elongation. It has demonstrated a significant reduction in axial elongation compared with single-vision lenses (1 year: −0.19 mm; 2 years: −0.28 mm) [11]. MFSCLs use a progressive or concentric ring design to create myopic defocus, resulting in a gradual reduction in the progression of myopia (1 year: 0.26 D; 2 years: 0.30 D; 3 years: 0.47 D) [11]. PPSLs aim to reduce hyperopic defocus in the peripheral retina, and researchers have reported a 52% slower progression of myopia compared with single-vision spectacles [11].

Although there is convincing evidence for the efficacy of optical interventions, questions persist, particularly regarding myopic rebound after the discontinuation of therapy. High discontinuation rates have been observed, reaching 15% for orthokeratology [12], 26% for MFSCLs [13], and 3% for PPSLs [14]. Given the substantial rebound effect in atropine treatment [15,16,17], it is plausible that optical interventions may yield similar results upon discontinuation. Several studies have explored post-treatment elongation or rebound effects [18,19]. In the realm of orthokeratology, opinions on the rebound effect vary, with research like DOEE [3] suggesting its presence. However, some propose the notion of faster progression in groups discontinuing orthokeratology [4] or attribute it to a regression to the mean effect [5]. Additionally, Swarbrick et al. [6] emphasize the rebound effect of orthokeratology through a novel study design. In contrast, soft contact lenses, particularly MiSight [7,8,9] and PPSLs [10,11], generally do not exhibit significant rebound effects. These preceding studies have resulted in varying opinions and underscore the need for a comprehensive understanding of the rebound effect and its associated factors in optical interventions.

In this study, we conduct a systematic review to address this knowledge gap, focusing on the reasons for the cessation of optical interventions for myopia control and the risks associated with discontinuation. The objective of this study is to equip practitioners with valuable insights, enabling them to provide patients and their guardians with thorough information regarding the potential risks of treatment discontinuation.

## 2. Materials and Methods

### 2.1. Study Design

In this systematic review, we aimed to investigate the potential rebound effects following the cessation of optical interventions for myopia control. To ensure transparency and minimize biases during the research process, we adhered strictly to the Preferred Reporting Items for Systematic Reviews and Meta-Analyses (PRISMA) statement [20]. The detailed methodology was predetermined and formally recorded on PROSPERO (CRD42023477253). Outcome measures included the axial length (AL) and spherical equivalent (SE).

### 2.2. Eligibility Criteria

To ensure the validity of our analysis, we included studies that met the following criteria: (1) randomized control trials (RCTs) or other interventional studies, (2) studies with data on AL or SE measurements, and (3) studies that enrolled children with myopia who underwent treatment for at least 6 months and subsequently ceased the intervention for more than 2 weeks.

### 2.3. Search Methods for Identifying Studies

Three authors (Yu-Chieh Chiu, Ping-Chiao Tsai, and Ssu-Hsien Lee) independently conducted a comprehensive search of PubMed, Cochrane CENTRAL, Embase, and ClinicalTrials.gov until 29 October 2023. Our search used the keywords “myopia OR nearsightedness” combined with “discontinue OR cease OR cessation OR stop OR stopped OR rebound OR swap OR swapped OR switch OR crossover.” There were no language limitations, and we thoroughly checked the reference lists for relevant studies.

### 2.4. Study Selection

Three authors, Yu-Chieh Chiu, Ping-Chiao Tsai, and Ssu-Hsien Lee, initially screened the titles and abstracts. Full articles were subsequently carefully analyzed to identify studies meeting the predetermined criteria. Any disagreements between the reviewers were resolved with a thorough discussion, and if needed, a fourth (or more) reviewer was involved to achieve a consensus.

### 2.5. Risk of Bias Assessment

To assess the methodological quality of the RCTs included in this analysis, we used the Cochrane risk-of-bias tool for randomized trials, version 2 (RoB 2). This tool comprises six main items related to randomization, intervention adherence, missing outcome data, outcome measurements, selective reporting, and the overall risk of bias. For other study designs, we used the Risk of Bias in Nonrandomized Studies of Interventions (ROBINS-I) tool, which assesses potential bias in the following seven domains: confounding, selection, intervention classification, deviations from intended interventions, missing data, outcome measurements, and reported results.

### 2.6. Data Extraction

Three authors (Yu-Chieh Chiu, Ping-Chiao Tsai, and Ssu-Hsien Lee) independently performed the data extraction from the included studies. Extracted data included demographic information, study design details, details of the myopia control devices, and measurements of the AL, SE, and other relevant outcomes. To avoid miscalculations, special attention was paid to accurately determine the treatment duration and cessation period in each trial. In cases in which the required data were not available in the published articles, the corresponding authors were contacted to obtain the original data.

### 2.7. Rebound Effect

The rebound effect is defined as an increase in the myopia progression measure in AL or SE during the discontinuation period compared with the treatment phase. It is classified into four levels based on changes in the AL or SE [21]. These levels are as follows: “no rebound effect” (an AL ≤ 0 mm/year or SE ≥ 0 D/year); “weak rebound effect” (an AL from 0 to 0.09 mm/year or SE from 0 to −0.25 D/year); “moderate rebound effect” (an AL from 0.09 to 0.18 mm/year or SE from −0.25 to −0.50 D/year); and “strong rebound effect” (an AL ≥ 0.18 mm/year or SE ≤ −0.50 D/year).

### 2.8. Cessation of Treatment

Participants should have discontinued the myopia-control treatment and refrained from using any myopia-control instrument during the washout period. The washout period was defined as a minimum of 2 weeks and involved either stopping the use of myopia-control device or transitioning to non-myopia control devices, such as single-vision spectacles or single-vision contact lenses.

### 2.9. Data Synthesis and Analysis

In this systematic review, we performed the data synthesis and analysis using statistical software, including Microsoft Excel 2019 and R (version 4.2.3). Our approach involved a straightforward data comparison and the use of descriptive statistics to investigate the potential rebound effect of myopia-control devices. We manually extracted data on changes in the axial length and spherical equivalent before and after the cessation of treatment from each of the included studies. We calculated the differences in myopia progression during the treatment and discontinuation periods. To standardize the presentation of the results, we expressed the progression of the AL and SE in standardized units per year.

## 3. Results

### 3.1. Literature Search

**Figure 1:**
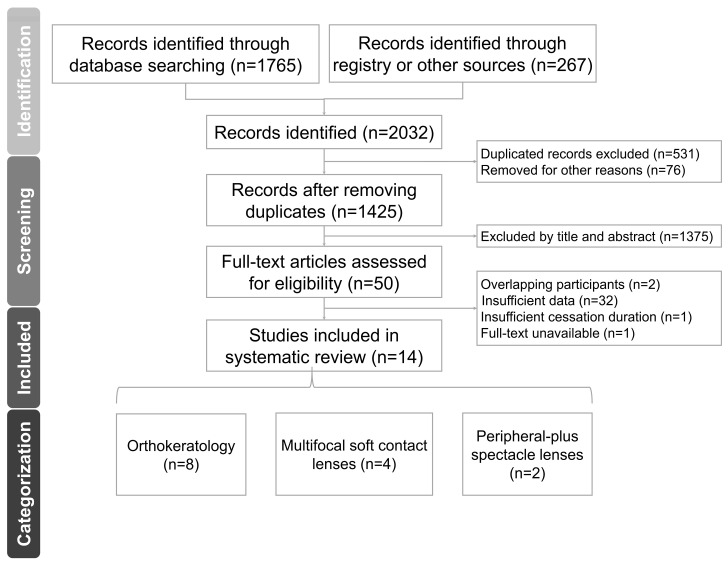
PRISMA flow chart.

Figure 1 outlines our search and selection processes. Initially, we conducted a thorough database search, yielding a total of 2032 studies. After we eliminated the duplicates, we reviewed the titles and abstracts, identifying 50 studies for full-text screening. Subsequently, we included 14 studies in our systematic review. Detailed information regarding the search keywords and the criteria for excluding specific studies can be found in Supplementary Tables S1 and S2.

### 3.2. Characteristics of Included Studies

Supplementary Table S3A–C provides the detailed characteristics of the included studies. Following a comprehensive search and literature review, we identified eight orthokeratology studies [3,6,12,13,14,15,16,17], four MFSCL studies [7,18,19,20], and two PPSL studies [10,11]. Our study included 703 participants, with an average age of 10.43 ± 1.96 years. The studies were primarily conducted in Asian countries, such as China and Hong Kong, with some performed in Spain and New Zealand, among others. Treatment durations ranged from 6 months to 2 years, and the cessation periods for orthokeratology varied from 2–3 weeks to 5 years, whereas studies in which MFSCLs and PPSLs were used consistently reported cessation periods of longer than 6 months.

### 3.3. Risk of Bias Assessment

To evaluate potential bias, we used the RoB 2 and ROBINS-I tools, and the findings are presented in Supplementary Tables S4 and S5. All nine RCTs [6,7,10,11,16,17,18,19,20] were rated as at a “high” risk of bias due to the absence of double blinding. Another notable observation was the unequal missing outcome data distribution between the two groups in some studies. Among the five non-RCTs assessed [3,12,13,14,15], nearly half were categorized as “critical” risk, followed by “moderate” risk, due to inadequate consideration of the confounding factors. It is worth noting that the majority of studies demonstrated a low bias risk in other domains.

### 3.4. Temporal Trend of Rebound Effect

**Figure 2:**
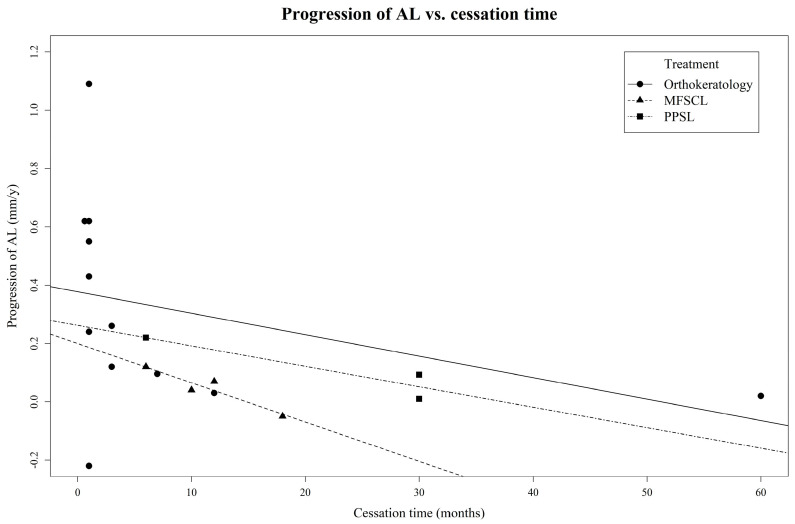
Time-dependent rebound effects on progression of axial-length myopia after cessation of optical interventions.

**Figure 3:**
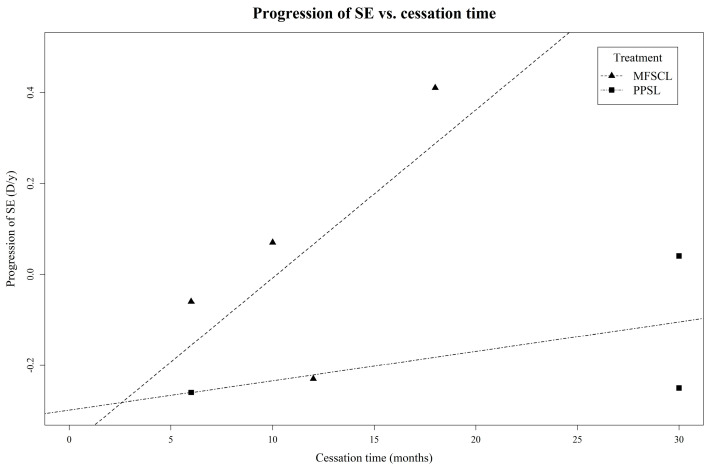
Time-dependent rebound effects on progression of spherical-equivalent myopia after cessation of optical interventions.

Our study confirms the temporal trend of the rebound effect in all three optical myopia control devices, as illustrated in Figure 2 and Figure 3. Table 1, Table 2 and Table 3 provide details on the rates of the myopia progression and rebound effects.

For orthokeratology, strong rebound effects were prevalent in studies with cessation periods of 1 month or less [6,12,13,15,17]. One study with a 3-month cessation period revealed strong–moderate rebound effects [2]. The cessation periods exceeding 6 months [3,14,16] revealed moderate rebound effects at 7 months [3] and weak rebound effects over 1 year [16].

MFSCLs displayed decreasing rebound effects over periods ranging from 6 months to 1.5 years [7,18,19,20], with one study featuring a 6-month cessation period showing a moderate rebound effect [20] and studies with cessation periods ranging from 6 months to 1 year indicating weak rebound effects [7,19]. For cessation periods longer than 1 year, no rebound effects were observed [18].

For PPSLs, a 6-month cessation duration showed a strong rebound effect [11], whereas a 2.5-year cessation period displayed moderate–weak rebound effects [10].

## 4. Discussion

This study is the first systematic review to investigate the rebound effects of optical interventions for myopia control. In a comprehensive review by Brennan et al. [21], the prevailing notion is that “rebound should be assumed until proven otherwise” in myopia control. In prior studies, orthokeratology yielded conflicting perspectives regarding the presence of a rebound effect, with some researchers suggesting its existence [3,6,22] and others arguing against it [4,5,9]. Conversely, soft contact lenses, specifically MiSight [7,8,9] and PPSLs [10,11], are generally thought to not exhibit rebound effects. Our research takes a distinct approach by avoiding a binary categorization of the rebound effect. In this pioneering, language-agnostic systematic review, we delve into the rebound effects of optical interventions for myopia control in children, revealing a nuanced temporal trend. This trend may be linked to the gradual reduction in the choroidal thickness and a decrease in myopic defocus on the retina. Factors such as a younger age, high baseline SE level, and high efficacy during the initial treatment period might contribute to the rebound effect after discontinuing optical interventions for myopia control.

### 4.1. Choroidal Thickness and Rebound Effect

Recent studies have drawn connections between myopia and choroidal thinning [23,24,25,26]. Furthermore, the efficacy of optical interventions has been linked to an increased choroidal thickness [13,27,28,29,30,31,32]. With regard to orthokeratology treatment, several studies have shown that the initial increase in the luminal area or the thickness of the large choroidal vessel play a role in slowing eye elongation [28,31]. 

Choroidal thickening is identified as an outcome of effective therapy [13,27,28,29,30,31,32]. Additionally, certain orthokeratology studies have explored changes in the choroidal thickness after treatment cessation, suggesting a potential return to baseline levels [13,32]. In a study conducted by Wang et al. [15] on orthokeratology, 106 patients were divided into AL-shortening and no-AL-shortening groups. As compared with baseline, the group with axial-length shortening showed shortened ALs at 1 month, indicating a potentially higher efficacy. Both groups exhibited strong rebound effects, with the AL-shortening group exhibiting relatively more robust rebound, possibly because of its higher efficacy, resulting in thicker choroids [15]. This observation aligns with the notion that the choroidal thickness can be an indicator of the effectiveness of orthokeratology [13,31].

For MFSCLs, the study conducted by Breher et al. [33] did not consider the choroidal thickness to be a primary factor contributing to the efficacy of myopia control. However, in a separate study led by Francisco et al. [34], responders (i.e., those with annual AL changes of <0.22 mm) using MiSight had significantly greater choroidal thicknesses than the nonresponders did. This suggests a link between the efficacy of MFSCLs and the choroidal thickness.

In the case of PPSLs, Haung et al. [35] highlight that spectacles equipped with aspherical lenslets demonstrated the ability to mitigate the reduction in the choroidal thickness after prolonged use, with the highly aspherical lenslets showing a more pronounced effect [35]. Moreover, choroidal thickening with defocus-incorporated multiple-segment spectacles has been observed to persist over a period of 2 years [36], coinciding with the effectiveness of defocus-incorporated multiple-segment lenses in slowing eye elongation. As expected, the removal of these myopia control devices results in the choroidal thickness reverting to its baseline [13,37,38].

In summary, the effectiveness of optical interventions for myopia control may be associated with choroidal thickening. Based on previous research, we propose that the discontinuation of these interventions might lead to a gradual reduction in the choroidal volume in the luminal area. This mechanism could be connected to the temporal trend of the rebound effect, suggesting a decline in efficacy and potential contribution to myopia progression. Further research is crucial to clarify the underlying mechanism and establish the causal relationship between the choroidal thickness and the rebound effect.

### 4.2. Peripheral Defocus on Retina and Rebound Effect

Previous studies have reported that myopic defocus stimulation induces cellular and biochemical changes in the retinal pigment epithelium, which can delay retinal growth signals to the sclera and ultimately influence the regulation of eye growth and the regression of myopia [39,40]. Orthokeratology, MFSCLs, and PPSLs have been used to introduce peripheral myopic defocus with the aim of slowing myopia progression [41]. Studies conducted on humans have shown that the human eye can detect the sign of imposed optical defocus and undergoes compensatory changes in the AL and choroidal thickness [33]. In addition to the increase in the choroidal thickness, incorporating a peripheral defocus design of optical intervention may also play a role in reducing axial elongation.

Orthokeratology lenses reshape the corneal epithelium, providing all-day myopic defocus. MFSCLs feature a concentric treatment and correction zone with 2.00 D of myopic retinal defocus for distant and near vision. PPSLs include a central optical zone for the correction of refractive errors, surrounded by segments for constant myopic defocus. In a study conducted by Delshad et al. [42], the authors measured changes in the eye length in response to both myopic and hyperopic defocus. The findings of their research indicated that the human eye undergoes elongation during hyperopic defocus and shortening during myopic defocus. Furthermore, the authors observed that significant changes in the AL occurred rapidly once the defocus stimulus was removed, which subsequently slowed over time [42]. This phenomenon may help explain the temporal trend in the rebound effect. Regardless of the mechanism, once the use of these three optical myopia-control devices is discontinued, there is a shift back to hyperopic defocus, with the most significant change occurring immediately after cessation.

### 4.3. Other Contributing Factors

Expanding on the mechanisms of choroidal thickening and peripheral defocus, individuals with high myopia undergoing orthokeratology may experience more pronounced rebound effects. Individuals with a higher baseline SE experience greater myopic defocus effects [43]. Notably, the choroidal thickness is greater in cases of high SEs [30]. After the discontinuation of wear, these two underlying mechanisms could potentially contribute to a more pronounced rebound effect. A study on orthokeratology involving 115 patients showed that the rebound effect may be more prominent in patients with myopia with high baseline SEs [12]. Moreover, another study highlighted that individuals with high myopia are particularly susceptible to this rebound phenomenon [44].

In addition to the refractive status, age can influence changes in the choroidal thickness, with younger children displaying more significant alterations [24]. Thus, discontinuing myopia control at a younger age may lead to a stronger rebound effect. Among the three studies that examined the use of MiSight [7,19,20], the study with a moderate rebound effect reported a baseline patient age of 10.8 ± 1.6 years [20], which is younger than the patients in the other two studies with no and weak rebound effects [7,19], in which the baseline ages of the patients were 13.2 ± 1.28 and 13.4 ± 0.85 years, respectively. Our findings align with previous research that has emphasized the importance of exercising caution when discontinuing the use of myopia-control soft contact lenses before the age of 13–14 years [8].

### 4.4. Adherence and Reasons for Cessation

#### 4.4.1. Orthokeratology

In real-world health care settings, patients may stop using orthokeratology lenses for various reasons. Reports indicate annual discontinuation rates ranging from 3% [45,46] to 9% [47], and, in some instances, reaching as high as 15% [48]. This dropout rate is consistent with other types of lenses, signifying the comparable discontinuation rates [5,49]. Lina Ma’s extensive 4-year study of 2499 patients reported 50 cases of lens discontinuation, which were attributed to nonadherence (50%), insufficient sleep (18.0%), financial constraints (16.0%), limited effectiveness (10.0%), and corneal infiltrates (6.0%) [50]. Importantly, there were no significant differences in the causes of discontinuation among the different age groups or genders. Interestingly, the study noted that individuals with high myopia were less likely to discontinue, possibly due to a stronger motivation for control.

#### 4.4.2. MFSCLs

The annual discontinuation rate for MFSCLs is approximately 17%–26% [18,51,52]. A significant challenge, accounting for 57% of the reasons for discontinuation, pertains to vision issues, particularly with multifocal lens designs compared with others, such as spherical and toric lenses [53]. This suggests that traditional measures of simple visual acuity may not fully capture the subjective performance. Moreover, some individuals endure discomfort silently without informing their doctors, even when the contact lenses are not suitable for them. Furthermore, discomfort ranks as the second most substantial challenge for new soft-contact-lens wearers, constituting 28% of the reported reasons [53]. In addition, the other challenges include inconvenience and loss of interest (23%), handling problems (21%), and cost (17%) [53].

#### 4.4.3. PPSLs

The dropout rate for PPSLs falls at approximately 2.1%, ranging from 1.6% to 3.0% [54]. The most common reason for the nontolerance of the spectacles is related to incorrect refraction, particularly inappropriate near-addition power, and difficulties adapting to bifocal or multifocal spectacles, making up 47.4% of the reasons. Communication errors contribute to 16.3% of the discontinuations, especially in cases involving language barriers between doctors and patients. Dispensing errors, such as providing spectacles with incorrect interpupillary distances or inappropriate lens types, account for 13.5% of the cases. In addition, nonadaptation problems account for 9.7%, data entry errors for 8.7%, errors related to binocular vision for 7.4%, and pathology-related errors for 6.4% [54]. These real-world challenges are often difficult to mitigate, resulting in instances of spectacle discontinuation.

### 4.5. Limitations

This study has several limitations that should be noted. First, the limited number of relevant studies and the fact that most were not originally designed to investigate the rebound effect resulted in a lack of standardized protocols and measurements. This constraint limited our ability to conduct subgroup analyses. Furthermore, grouping different types of lens designs, such as MiSight and positive spherical aberration SCLs, under the category of MFSCLs may introduce variations in the comfort and ease of adaptation for the wearer, which can influence compliance and the decision to discontinue. Finally, orthokeratology induces corneal redistribution and remodeling [55,56,57], causing the cornea to rebound to its initial shape postcessation [58], which may potentially affect the AL. Fortunately, the corneal thickness returned to baseline values within 2 weeks of discontinuation [59], whereas the depth of the anterior chamber remained stable [17]. To minimize confounding, we excluded research with cessation periods shorter than 2 weeks.

## 5. Conclusions

In conclusion, our study underscores that discontinuing optical interventions for myopia control in children can lead to rebound effects. Specifically, we observed a robust rebound effect in orthokeratology, a less pronounced rebound effect in MFSCLs, and a variable rebound effect in PPSLs. Our analysis indicates a temporal pattern in the rebound effects of these interventions, likely linked to the gradual reversal of choroidal thickening and the disappearance of peripheral myopic defocus. These findings emphasize the intricate nature of rebound effects in myopia-control strategies. Future well-designed studies, prioritizing rebound effects as the primary outcomes and incorporating comparative analyses across different treatments and cessation durations, are imperative to refine optimal treatment and cessation strategies. Additionally, comprehensive research is essential to fully elucidate the precise underlying mechanisms.

## Figures and Tables

**Table 1:** Axial-length myopia progression after cessation of orthokeratology.

Study	N	Age (years)	Treatment/Cessation Time	Treatment Progression Rate of AL (mm/year)	Cessation Progression Rate of AL (mm/year)	Rebound Effect
Swarbrick HA [6]	13	13.4 ± 1.9	6 m/2–3 w	−0.04 ± 0.18	0.58 ± 1.54	Strong
Li Z [13]	29	12.3 ± 1.7	12 m/1 m	0.17 ± 0.16	0.72 ± 2.04	Strong
Wang A [15]	ALS, 54	9.6 ± 1.3	19 m/1 m	0.11 ± 0.11	1.20 ± 0.60	Strong
	NALS, 52	9.1 ± 1.4		0.29 ± 0.16	0.72 ± 0.60	Strong
Zhu Q [17]	142	9.2 ± 1.3	12 m/1 m	0.22 ± 0.68	0.00 ± 8.16	None
Li H [12]	Low SE, 59	8.0–16.0 *	1 y/1 m	0.12 ± 0.63	0.36 ± 7.42	Strong
	High SE, 56			0.10 ± 0.73	0.72 ± 8.62	Strong
	Low SE, 59		1 y/3 m	0.12 ± 0.63	0.24 ± 2.44	Moderate
	High SE, 56			0.10 ± 0.73	0.36 ± 2.96	Strong
Cho P [3]	15	10.0 (10.0–14.0) *	2 y/7 m	0.17 ± 0.46	0.26 ± 0.14	Moderate
Wei S [16]	45	11.0 ± 1.9	1 y/1 y	0.20 ± 0.26	0.23 ± 0.17	Weak
Santodomingo-Rubido J [14]	8	10.4 ± 0.5	2 y/5 y	0.14 ± 0.11	0.16 ± 0.11	Weak

Note: w: week; m: month; y: year; mm: millimeter. ALS, NALS: Wang et al. [15] categorized patients into axial-length-shortening (ALS) and no-axial-length-shortening (NALS) groups, with the ALS group including patients with shortened axial lengths compared with baseline at a one-month visit, data were adapted from Wang et al. [15], 2020. Low SE, High SE: Li et al. [12] divided patients into a low-spherical-equivalent group (SE ≤ −4.0 D) and high-spherical-equivalent group (from −4.0 to −6.0 D), data were adapted from Li et al. [12], 2021. All data shown are means ± SDs, if not marked otherwise; * data shown in range.

**Table 2:** Myopia progression of axial length and spherical equivalent after cessation of the use of multifocal soft contact lenses.

Study	N	Age (years)	Treatment/Cessation Time	Treatment Progression Rate of AL (mm/year)	Cessation Progression Rate of AL (mm/year)	Rebound Effect	Treatment Progression Rate of SE (D/year)	Cessation Progression Rate of SE (D/year)	Rebound Effect
Weng [20]	21	10.8 ± 1.6	6 m/6 m	0.16 ± 0.16	0.28 ± 0.16	Moderate	−0.46 ± 0.66	−0.52 ± 0.36	Weak
Anstice [19]	34	13.4 ± 0.9	10 m/10 m	0.13± 0.10	0.17 ± 0.11	Weak	−0.53 ± 0.40	−0.46 ± 0.46	None
Ruiz-Pomeda [7]	18	13.2 ± 1.3	2 y/1 y	0.15 ± 0.04	0.22 ± 0.11	Weak	−0.24 ± 0.09	−0.46 ± 0.39	Weak
Cheng [18]	39	9.7 ± 1.1	2 y/1.5 y	0.24	0.19	No	−0.67	−0.26	None

**Table 3:** Myopia progression of axial length and spherical equivalent after cessation of peripheral-plus spherical lenses.

Study	N	Age (years)	Treatment/Cessation Time	Treatment Progression Rate of AL (mm/y)	Cessation Progression Rate of AL (mm/y)	Rebound Effect	Treatment Progression Rate of SE (D/y)	Cessation Progression Rate of SE (D/y)	Rebound Effect
Sankaridurg [11]	50	11.2 ± 1.6	6 m/6 m	0.12 ± 0.30	0.34 ± 0.26	Strong	−0.40 ± 0.62	−0.66 ± 0.54	Moderate
Lam [10]	14	10.2 ± 1.5	3.5 y/2.5 y	0.11 ± 0.10	0.12 ± 0.08	Weak	−0.23 ± 0.59	−0.19 ± 0.15	None
	18	10.3 ± 1.7	1.5 y/2.5 y	0.03 ± 0.15	0.12 ± 0.08	Moderate	−0.01 ± 0.67	−0.25 ± 0.20	Weak

## Data Availability

Data are contained within the article or Supplementary Materials. Further inquiries can be directed to the corresponding author.

## Supplementary Materials

The following supporting information can be downloaded at https://www.mdpi.com/article/10.3390/jcm13010053/s1, Table S1: Keywords and search results in different databases; Table S2 Excluded studies and reasons [32,44,49,53,60,61,62,63,64,65,66,67,68,69,70,71,72,73,74,75,76,77,78,79,80,81,82,83,84,85,86,87,88,89]; Table S3A–3C: Characteristics of included studies of orthokeratology [3,6,12,13,14,15,16,17], characteristics of included studies of multifocal soft contact lenses [7,18,19,20], and characteristics of included studies of peripheral-plus spectacle lenses [10,11]; Table S4: Detailed quality assessment of included studies using Cochrane risk-of-bias 2 tool [6,7,10,11,16,17,18,19,20]; Table S5: Detailed quality assessment of included studies using the Cochrane Risk Of Bias In Nonrandomized Studies of Interventions [3,12,13,14,15].

## References

[1] Dolgin E. (2015). The myopia boom. Nature.

[2] Holden B.A., Fricke T.R., Wilson D.A., Jong M., Naidoo K.S., Sankaridurg P., Wong T.Y., Naduvilath T., Resnikoff S. (2016). Global Prevalence of Myopia and High Myopia and Temporal Trends from 2000 through 2050. Ophthalmology.

[3] Cho P., Cheung S.W. (2017). Discontinuation of orthokeratology on eyeball elongation (DOEE). Cont. Lens Anterior Eye.

[4] Cho P., Tan Q. (2019). Myopia and orthokeratology for myopia control. Clin. Exp. Optom..

[5] Bullimore M.A., Johnson L.A. (2020). Overnight orthokeratology. Cont. Lens Anterior Eye.

[6] Swarbrick H.A., Alharbi A., Watt K., Lum E., Kang P. (2015). Myopia control during orthokeratology lens wear in children using a novel study design. Ophthalmology.

[7] Ruiz-Pomeda A., Prieto-Garrido F.L., Hernández Verdejo J.L., Villa-Collar C. (2021). Rebound Effect in the Misight Assessment Study Spain (Mass). Curr. Eye Res..

[8] Sankaridurg P., Berntsen D.A., Bullimore M.A., Cho P., Flitcroft I., Gawne T.J., Gifford K.L., Jong M., Kang P., Ostrin L.A. (2023). IMI 2023 Digest. Investig. Ophthalmol. Vis. Sci..

[9] Logan N.S., Bullimore M.A. (2023). Optical interventions for myopia control. Eye.

[10] Lam C.S.Y., Tang W.C., Zhang H.Y., Lee P.H., Tse D.Y.Y., Qi H., Vlasak N., To C.H. (2023). Long-term myopia control effect and safety in children wearing DIMS spectacle lenses for 6 years. Sci. Rep..

[11] Sankaridurg P., Weng R., Tran H., Spiegel D.P., Drobe B., Ha T., Tran Y.H., Naduvilath T. (2023). Spectacle Lenses with Highly Aspherical Lenslets for Slowing Myopia: A Randomized, Double-Blind, Cross-Over Clinical Trial: Parts of these data were presented as a poster at the Annual Research in Vision and Ophthalmology meeting, 2022. Am. J. Ophthalmol..

[12] Li H., Ouyang J. (2019). The effect of long-term wearing of keratoplasty lenses and stopping wearing on corneal thickness. Proceeding Clin. Med..

[13] Li Z., Hu Y., Cui D., Long W., He M., Yang X. (2019). Change in subfoveal choroidal thickness secondary to orthokeratology and its cessation: A predictor for the change in axial length. Acta Ophthalmol..

[14] Santodomingo-Rubido J., Villa-Collar C., Gilmartin B., Gutiérrez-Ortega R., Sugimoto K. (2017). Long-term Efficacy of Orthokeratology Contact Lens Wear in Controlling the Progression of Childhood Myopia. Curr. Eye Res..

[15] Wang A., Yang C., Shen L., Wang J., Zhang Z., Yang W. (2022). Axial length shortening after orthokeratology and its relationship with myopic control. BMC Ophthalmol..

[16] Wei S., Li S., Sun Y., Kang M., Wang J., Ran A., Zhang F. (2017). Effects of orthokeratology lenses on ocular biometric parameters in children with low to moderate myopia. Chin. J. Optom. Ophthalmol. Vis. Sci..

[17] Zhu Q., Yin J., Li X., Hu M., Xue L., Zhang J., Zhou Y., Zhang X., Zhu Y., Zhong H. (2023). Effects of Long-Term Wear and Discontinuation of Orthokeratology Lenses on the Eyeball Parameters in Children with Myopia. Int. J. Med. Sci..

[18] Cheng X., Xu J., Chehab K., Exford J., Brennan N. (2016). Soft Contact Lenses with Positive Spherical Aberration for Myopia Control. Optom. Vis. Sci..

[19] Anstice N.S., Phillips J.R. (2011). Effect of dual-focus soft contact lens wear on axial myopia progression in children. Ophthalmology.

[20] Weng R., Lan W., Bakaraju R., Conrad F., Naduvilath T., Yang Z., Sankaridurg P. (2022). Efficacy of contact lenses for myopia control: Insights from a randomised, contralateral study design. Ophthalmic Physiol. Opt..

[21] Brennan N.A., Toubouti Y.M., Cheng X., Bullimore M.A. (2021). Efficacy in myopia control. Prog. Retin. Eye Res..

[22] VanderVeen D.K., Kraker R.T., Pineles S.L., Hutchinson A.K., Wilson L.B., Galvin J.A., Lambert S.R. (2019). Use of Orthokeratology for the Prevention of Myopic Progression in Children: A Report by the American Academy of Ophthalmology. Ophthalmology.

[23] Bin Wei W., Xu L., Jonas J.B., Shao L., Du K.F., Wang S., Chen C.X., Xu J., Wang Y.X., Zhou J.Q. (2013). Subfoveal choroidal thickness: The Beijing Eye Study. Ophthalmology.

[24] Xiong S., He X., Zhang B., Deng J., Wang J., Lv M., Zhu J., Zou H., Xu X. (2020). Changes in Choroidal Thickness Varied by Age and Refraction in Children and Adolescents: A 1-Year Longitudinal Study. Am. J. Ophthalmol..

[25] Tian F., Zheng D., Zhang J., Liu L., Duan J., Guo Y., Wang Y., Wang S., Sang Y., Zhang X. (2021). Choroidal and Retinal Thickness and Axial Eye Elongation in Chinese Junior Students. Investig. Ophthalmol. Vis. Sci..

[26] Jin W.Q., Huang S.H., Jiang J., Mao X.J., Shen M.X., Lian Y. (2018). Short term effect of choroid thickness in the horizontal meridian detected by spectral domain optical coherence tomography in myopic children after orthokeratology. Int. J. Ophthalmol..

[27] Chen Z., Xue F., Zhou J., Qu X., Zhou X. (2016). Effects of Orthokeratology on Choroidal Thickness and Axial Length. Optom. Vis. Sci..

[28] Li Z., Cui D., Hu Y., Ao S., Zeng J., Yang X. (2017). Choroidal thickness and axial length changes in myopic children treated with orthokeratology. Cont. Lens Anterior Eye.

[29] Zhu Q., Zhao Q. (2022). Short-term effect of orthokeratology lens wear on choroidal blood flow in children with low and moderate myopia. Sci. Rep..

[30] Wang Z., Chen J., Kang J., Niu T., Guo L., Fan L. (2023). Axial Length Control Is Associated with a Choroidal Thickness Increase in Myopic Adolescents after Orthokeratology. Eye Contact Lens.

[31] Wu H., Peng T., Zhou W., Huang Z., Li H., Wang T., Zhang J., Zhang K., Li H., Zhao Y. (2023). Choroidal vasculature act as predictive biomarkers of long-term ocular elongation in myopic children treated with orthokeratology: A prospective cohort study. Eye Vis..

[32] Lau J.K., Wan K., Cheung S.W., Vincent S.J., Cho P. (2019). Weekly Changes in Axial Length and Choroidal Thickness in Children During and Following Orthokeratology Treatment with Different Compression Factors. Transl. Vis. Sci. Technol..

[33] Breher K., García García M., Ohlendorf A., Wahl S. (2018). The effect of the optical design of multifocal contact lenses on choroidal thickness. PLoS ONE.

[34] Prieto-Garrido F.L., Villa-Collar C., Hernandez-Verdejo J.L., Alvarez-Peregrina C., Ruiz-Pomeda A. (2022). Changes in the Choroidal Thickness of Children Wearing MiSight to Control Myopia. J. Clin. Med..

[35] Huang Y., Li X., Wu J., Huo J., Zhou F., Zhang J., Yang A., Spiegel D.P., Chen H., Bao J. (2022). Effect of spectacle lenses with aspherical lenslets on choroidal thickness in myopic children: A 2-year randomised clinical trial. Br. J. Ophthalmol..

[36] Lam C.S.Y., Tang W.C., Tse D.Y.-Y., Lee R.P.K., Chun R.K.M., Hasegawa K., Qi H., Hatanaka T., To C.H. (2020). Defocus Incorporated Multiple Segments (DIMS) spectacle lenses slow myopia progression: A 2-year randomised clinical trial. Br. J. Ophthalmol..

[37] Chiang S.T., Phillips J.R., Backhouse S. (2015). Effect of retinal image defocus on the thickness of the human choroid. Ophthalmic Physiol. Opt..

[38] Wang D., Chun R.K.M., Liu M., Lee R.P.K., Sun Y., Zhang T., Lam C., Liu Q., To C.H. (2016). Optical Defocus Rapidly Changes Choroidal Thickness in Schoolchildren. PLoS ONE.

[39] Troilo D., Smith E.L., Nickla D.L., Ashby R., Tkatchenko A.V., Ostrin L.A., Gawne T.J., Pardue M.T., Summers J.A., Kee C.S. (2019). IMI—Report on Experimental Models of Emmetropization and Myopia. Investig. Ophthalmol. Vis. Sci..

[40] Rymer J., Wildsoet C.F. (2005). The role of the retinal pigment epithelium in eye growth regulation and myopia: A review. Vis. Neurosci..

[41] Wolffsohn J.S., Whayeb Y., Logan N.S., Weng R. (2023). IMI-Global Trends in Myopia Management Attitudes and Strategies in Clinical Practice-2022 Update. Investig. Ophthalmol. Vis. Sci..

[42] Delshad S., Collins M.J., Read S.A., Vincent S.J. (2020). The time course of the onset and recovery of axial length changes in response to imposed defocus. Sci. Rep..

[43] Hiraoka T., Kakita T., Okamoto F., Oshika T. (2015). Influence of ocular wavefront aberrations on axial length elongation in myopic children treated with overnight orthokeratology. Ophthalmology.

[44] Xing X.H., Wang X.Y., Wang S.H., Tu J. (2023). Influence of the duration of orthokeratology lens cessation on patients’ refractive status and corneal endothelial cells. Int. Eye Sci..

[45] Santodomingo-Rubido J., Villa-Collar C., Gilmartin B., Gutiérrez-Ortega R. (2012). Orthokeratology vs. spectacles: Adverse events and discontinuations. Optom. Vis. Sci..

[46] Cho P., Cheung S.W., Edwards M. (2005). The longitudinal orthokeratology research in children (LORIC) in Hong Kong: A pilot study on refractive changes and myopic control. Curr. Eye Res..

[47] Kakita T., Hiraoka T., Oshika T. (2011). Influence of overnight orthokeratology on axial elongation in childhood myopia. Investig. Ophthalmol. Vis. Sci..

[48] Walline J.J., Jones L.A., Sinnott L.T. (2009). Corneal reshaping and myopia progression. Br. J. Ophthalmol..

[49] Gispets J., Yébana P., Lupón N., Cardona G., Pérez-Corral J., Pauné J., Cortilla B. (2022). Efficacy, predictability and safety of long-term orthokeratology: An 18-year follow-up study. Cont. Lens Anterior Eye.

[50] Ma L., Xu M., Wang J., Niu X. (2022). Analysis of the Reasons for the Discontinuation of Orthokeratology Lens Use: A 4-Year Retrospective Study. Eye Contact Lens.

[51] Sulley A., Young G., Hunt C., McCready S., Targett M.T., Craven R. (2018). Retention Rates in New Contact Lens Wearers. Eye Contact Lens.

[52] Sulley A., Young G., Hunt C. (2017). Factors in the success of new contact lens wearers. Cont. Lens Anterior Eye.

[53] Morgan P.B., Sulley A.L. (2023). Challenges to the new soft contact lens wearer and strategies for clinical management. Cont. Lens Anterior Eye.

[54] Bist J., Kaphle D., Marasini S., Kandel H. (2021). Spectacle non-tolerance in clinical practice—A systematic review with meta-analysis. Ophthalmic Physiol. Opt..

[55] Swarbrick H.A., Wong G., O’Leary D.J. (1998). Corneal response to orthokeratology. Optom. Vis. Sci..

[56] Alharbi A., Swarbrick H.A. (2003). The effects of overnight orthokeratology lens wear on corneal thickness. Investig. Ophthalmol. Vis. Sci..

[57] Lian Y., Shen M., Jiang J., Mao X., Lu P., Zhu D., Chen Q., Wang J., Lu F. (2013). Vertical and horizontal thickness profiles of the corneal epithelium and Bowman’s layer after orthokeratology. Investig. Ophthalmol. Vis. Sci..

[58] Yeh T.N., Green H.M., Zhou Y., Pitts J., Kitamata-Wong B., Lee S., Wang S.L., Lin M.C. (2013). Short-term effects of overnight orthokeratology on corneal epithelial permeability and biomechanical properties. Investig. Ophthalmol. Vis. Sci..

[59] Wan K., Yau H.T., Cheung S.W., Cho P. (2021). Corneal thickness changes in myopic children during and after short-term orthokeratology lens wear. Ophthalmic Physiol. Opt..

[60] Santodomingo-Rubido J., Villa-Collar C., Gilmartin B., Gutiérrez-Ortega R. (2014). Short-term changes in ocular biometry and refraction after discontinuation of long-term orthokeratology. Eye Contact Lens.

[61] Polse K.A., Brand R.J., Schwalbe J.S., Vastine D.W., Keener R.J. (1983). The Berkeley Orthokeratology Study, Part II: Efficacy and duration. Am. J. Optom. Physiol. Opt..

[62] Huang Y., Li X., Ding C., Chen Y., Chen H., Bao J. (2022). Orthokeratology reshapes eyes to be less prolate and more symmetric. Cont. Lens Anterior Eye.

[63] Polse K.A., Brand R.J., Vastine D.W., Schwalbe J.S. (1983). Corneal change accompanying orthokeratology. Plastic or elastic? Results of a randomized controlled clinical trial. Arch. Ophthalmol..

[64] Wu R., Stapleton F., Swarbrick H.A. (2009). Residual corneal flattening after discontinuation of long-term orthokeratology lens wear in asian children. Eye Contact Lens.

[65] Yang L., Guo X., Xie P. (2015). Observation of orthokeratology discontinuation. Zhonghua Yan Ke Za Zhi.

[66] Zhao L., Jing L., Li J., Du X. (2022). Changes in corneal densitometry after long-term orthokeratology for myopia and short-term discontinuation. PLoS ONE.

[67] Guo Y., Liu L., Peng L., Fu J., Guo W., Tang P. (2023). Effect of overnight orthokeratology lenses on tear film stability in children. Cont. Lens Anterior Eye.

[68] Nieto-Bona A., González-Mesa A., Nieto-Bona M.P., Villa-Collar C., Lorente-Velázquez A. (2011). Long-term changes in corneal morphology induced by overnight orthokeratology. Curr. Eye Res..

[69] Nakamura Y., Hieda O., Yokota I., Teramukai S., Sotozono C., Kinoshita S. (2021). Comparison of myopia progression between children wearing three types of orthokeratology lenses and children wearing single-vision spectacles. Jpn. J. Ophthalmol..

[70] Lum E., Golebiowski B., Swarbrick H.A. (2017). Changes in corneal subbasal nerve morphology and sensitivity during orthokeratology: Recovery of change. Ocul. Surf..

[71] Chen Z., Zhou J., Xue F., Zhou X., Qu X. (2018). Increased Corneal Toricity after Long-Term Orthokeratology Lens Wear. J. Ophthalmol..

[72] Wan K., Lau J.K., Cheung S.W., Cho P. (2020). Refractive and corneal responses of young myopic children to short-term orthokeratology treatment with different compression factors. Cont. Lens Anterior Eye.

[73] Kang P., Swarbrick H. (2017). Discontinuation of long term orthokeratology lens wear and subsequent refractive surgery outcome. Cont. Lens Anterior Eye.

[74] Cho P., Cheung S.W. (2012). Retardation of myopia in Orthokeratology (ROMIO) study: A 2-year randomized clinical trial. Investig. Ophthalmol. Vis. Sci..

[75] Soni P.S., Nguyen T.T., Bonanno J.A. (2004). Overnight orthokeratology: Refractive and corneal recovery after discontinuation of reverse-geometry lenses. Eye Contact Lens.

[76] Kobayashi Y., Yanai R., Chikamoto N., Chikama T., Ueda K., Nishida T. (2008). Reversibility of effects of orthokeratology on visual acuity, refractive error, corneal topography, and contrast sensitivity. Eye Contact Lens.

[77] Hiraoka T., Okamoto C., Ishii Y., Okamoto F., Oshika T. (2009). Recovery of corneal irregular astigmatism, ocular higher-order aberrations, and contrast sensitivity after discontinuation of overnight orthokeratology. Br. J. Ophthalmol..

[78] Dong J., Liu Z.Y., Feng Y.L. (2013). The comparative study of orthokeratology lens and frame glasses in adolescent myopia. Chin. J. Strabismus Pediatr. Ophthalmol..

[79] Jin Y.M., Zhang Y., Li H., Hu B.Y. (2011). Effects of Orthokeratology Lens on Corneal Endothelium and Corneal Thickness. Med. J. PUMCH.

[80] Mai Z.C., Lin Z.X. (2016). Study on the Changes of Refraction and Corneal Shape after Stop Wearing Orthokeratology. Mod. Diagn. Treat..

[81] Zhou Z.X., Xu S.S., Yi S.P. (2016). Clinical effect of orthokeratology for juvenile with myopia astigmatism and its effects on corneaI endotheliaI cells. Int. Eye Sci..

[82] Lu W.W., Lian Y., Yu X.L., Jin W.Q. (2019). Changes and Relationship Analysis of Ocular Parameters After Short-term Discontinuation of Orthokeratology. J. Sun Yat-Sen Univ. (Med. Sci.).

[83] Hu J., Geng L., Yang Q.H., Wamg L.Q., Huang Y.F. (2019). Analysis of corneal morphology after one-month discontinuation of orthokeratology treatment. Acad. J. Chin. PLA Med. Sch..

[84] Sibug M.E., Datiles M.B., Kashima K., McCain L., Kracher G. (1991). Specular microscopy studies on the corneal endothelium after cessation of contact lens wear. Cornea.

[85] Lei Y., Zheng X., Hou J., Xu B., Mu G. (2015). Effects of long-term soft contact lens wear on the corneal thickness and corneal epithelial thickness of myopic subjects. Mol. Med. Rep..

[86] Sankaridurg P. (2017). Contact lenses to slow progression of myopia. Clin. Exp. Optom..

[87] Zhu Q., Liu Y., Tighe S., Zhu Y., Su X., Lu F., Hu M. (2019). Retardation of Myopia Progression by Multifocal Soft Contact Lenses. Int. J. Med. Sci..

[88] Ruiz-Pomeda A., Pérez-Sánchez B., Prieto-Garrido F.L., Gutiérrez-Ortega R., Villa-Collar C. (2018). MiSight Assessment Study Spain: Adverse Events, Tear Film Osmolarity, and Discontinuations. Eye Contact Lens.

[89] Berntsen D.A., Mutti D.O., Zadnik K. (2010). Study of Theories about Myopia Progression (STAMP) design and baseline data. Optom. Vis. Sci..

