# Supplementary material for: Systematic Review of Myopia Progression after Cessation of Optical Interventions for Myopia Control
Source: J Clin Med. 2023 Dec 21;13(1):53. doi: 10.3390/jcm13010053 (PMC10779574; doi:10.3390/jcm13010053)
Supplement: Supplementary file 1 [file jcm-13-00053-s001.zip › jcm-2756785-supplementary.pdf]

Supplementary Material

Systematic Review of Myopia Progression after Cessation of Optical Interventions for Myopia Control

Yu-Chieh Chiu [1,†], Ping-Chiao Tsai [1,†], Ssu-Hsien Lee [1,†], Jen-Hung Wang [2] and Cheng-Jen Chiu [3,4,*]

1 School of Medicine, Tzu Chi University, Hualien 970, Taiwan; hl2200571@tzuchi.com.tw (Y.-C.C.); hl2200544@tzuchi.com.tw (P.-C.T.); hl2200543@tzuchi.com.tw (S.-H.L.)

2 Department of Medical Research, Buddhist Tzu Chi General Hospital, Hualien 970, Taiwan;

paulwang@tzuchi.com.tw

3 Department of Ophthalmology and Visual Science, Tzu Chi University, Hualien 970, Taiwan

4 Department of Ophthalmology, Hualien Tzu Chi Hospital, the Buddhist Tzu Chi Medical Foundation, Hualien 970, Taiwan

* Correspondence: drcjchiu@tzuchi.com.tw

† These authors contributed equally to this work.

Table S1. Keywords and search results in different databases

Database	Keyword	Filter	Date	Results
PubMed	(myopia OR nearsightedness) AND (discontinue OR cease OR cessation OR stop OR stopped OR rebound OR swap OR swapped OR switch OR crossover)	NA	October 29, 2023	634
Embase	((myopia.mp. OR exp myopia/) OR nearsightedness.mp.) AND (discontinue.mp. OR cease.mp. OR cessation.mp. OR stop.mp. OR stopped.mp. OR (rebound.mp. OR exp rebound/) OR swap.mp. OR swapped.mp. OR switch.mp. OR crossover.mp.)	NA	October 29, 2023	582
Cochrane CENTRAL	((myopia OR [myopia]) OR nearsightedness) AND (discontinue OR cease OR cessation OR stop OR stopped OR rebound OR swap OR swapped OR switch OR crossover)	NA	October 29, 2023	294
ClinicalTrials.gov	(myopia OR nearsightedness) AND (discontinue OR cease OR cessation OR stop OR stopped OR rebound OR swap OR swapped OR switch OR crossover)	Other Terms Condition or disease	October 29, 2023	255

NA: not applied

Table S2. Excluded studies and reasons

Study	Reason for exclusion
Short-term changes in ocular biometry and refraction after discontinuation of long-term orthokeratology (1)	Insufficient cessation duration
Weekly Changes in Axial Length and Choroidal Thickness in Children During and Following Orthokeratology Treatment With Different Compression Factors (2)	Insufficient treatment duration
The Berkeley Orthokeratology Study, Part II: Efficacy and duration (3)	Insufficient data
Orthokeratology reshapes eyes to be less prolate and more symmetric (4)	Insufficient data
Corneal change accompanying orthokeratology. Plastic or elastic? Results of a randomized controlled clinical trial (5)	Insufficient data
Residual corneal flattening after discontinuation of long-term orthokeratology lens wear in asian children (6)	Insufficient data
Observation of orthokeratology discontinuation (7)	No full text available
Changes in corneal densitometry after long-term orthokeratology for myopia and short-term discontinuation (8)	Insufficient data
Effect of overnight orthokeratology lenses on tear film stability in children (9)	Insufficient data

Long-term changes in corneal morphology induced by overnight orthokeratology (10)	Insufficient data
Comparison of myopia progression between children wearing three types of orthokeratology lenses and children wearing single-vision spectacles (11)	Insufficient data
Changes in corneal subbasal nerve morphology and sensitivity during orthokeratology: Recovery of change (12)	Insufficient data
Increased Corneal Toricity after Long-Term Orthokeratology Lens Wear (13)	Insufficient data
Refractive and corneal responses of young myopic children to short-term orthokeratology treatment with different compression factors (14)	Insufficient data
Corneal thickness changes in myopic children during and after short-term orthokeratology lens wear (15)	Insufficient data
Discontinuation of long term orthokeratology lens wear and subsequent refractive surgery outcome (16)	Insufficient data
Efficacy, predictability and safety of long-term orthokeratology: An 18-year follow-up study (17)	Insufficient data
Retardation of myopia in Orthokeratology (ROMIO) study: a 2-year randomized clinical trial (18)	Insufficient data
Overnight orthokeratology: refractive and corneal recovery after discontinuation of reverse-geometry lenses (19)	Insufficient data
Reversibility of effects of orthokeratology on visual acuity, refractive error, corneal topography, and contrast sensitivity (20)	Insufficient data

Recovery of corneal irregular astigmatism, ocular higher-order aberrations, and contrast sensitivity after discontinuation of overnight orthokeratology (21)	Insufficient data
Influence of the duration of orthokeratology lens cessation on patients’ refractive status and corneal endothelial cells (22)	Insufficient data
The comparative study of orthokeratology lens and frame glasses in adolescent myopia (23)	Insufficient data
Effects of Orthokeratology Lens on Corneal Endothelium and Corneal Thickness (24)	Insufficient data
Study on the Changes of Refraction and Corneal Shape after Stop Wearing Orthokeratology (25)	Insufficient data
Clinical effect of orthokeratology for juvenile with myopia astigmatism and its effects on corneaI endothelial cells (26)	Insufficient data
Observation of orthokeratology discontinuation (27)	Insufficient data
Changes and Relationship Analysis of Ocular Parameters After Short-term Discontinuation of Orthokeratology (28)	Insufficient data
Analysis of corneal morphology after one-month discontinuation of orthokeratology treatment (29)	Insufficient data
Specular microscopy studies on the corneal endothelium after cessation of contact lens wear (30)	Insufficient data
Effects of long-term soft contact lens wear on the corneal thickness and corneal epithelial thickness of myopic subjects (31)	Insufficient data

Contact lenses to slow progression of myopia (32)	Insufficient data
Retardation of Myopia Progression by Multifocal Soft Contact Lenses (33)	Insufficient data
Challenges to the new soft contact lens wearer and strategies for clinical management (34)	Insufficient data
MiSight Assessment Study Spain: Adverse Events, Tear Film Osmolarity, and Discontinuations (35)	Overlapping participants, insufficient data
Study of Theories about Myopia Progression (STAMP) design and baseline data, discontinuation (36)	Overlapping participants

Table S3A. Characteristic of included studies of orthokeratology

First Author	Year	Study Design	(Group), N	Age	Baseline AL (mm)	Baseline SE (D)	Male: Female	Treatment duration	Cessation duration	Country	COI
Swarbrick HA (37)	2014	RCT	13	13.4 ± 1.9	NA	-2.43 ± 0.98	14:12	1y	2w	East Asian	Y
Cho P (38)	2016	Non-RCT	15	10.0 (10–14)	24.61 ± 0.90<br>24.07 ± 0.79	-2.36 ± 1.06	NA	2y	14m	Hong Kong	Y
Santodomingo-Rubido J (39)	2016	Non-RCT	8	10.4 ± 0.5	24.66 ± 0.30	Sphere: -2.31 ± 0.38<br>Cylinder: -0.38 ± 0.08	4:4	2y	5y	Spain	Y
Wei S (40)	2017	RCT	45	11.0 ± 1.9	25.0 ± 0.8	-3.42 ± 1.36	17:28	1y	1m	China	Y
Li H (41)	2019	Non-RCT	Low SE: 59	8-16	25.06 ± 0.62	A <= -4	28:31	1y	3m	China	N
			High SE: 56		25.62 ± 0.71	B -4 ~ -6	27:29				
Li Z (42)	2019	Non-RCT	29	12.31 ± 1.71	25.18 ± 0.77	-3.16 ± 0.85	13:16	1y	1m	China	N
Wang A (43)	2022	Non-RCT	ALS Group: 54	9.63 ± 1.34	24.68 ± 0.9	-2.98 1.25	22:32	19m	1m	China	Y
			NALS Group: 52	9.12 ± 1.41	24.50 ± 0.68	-2.6 1.02	19:33				

Zhu Q (44)	2023	RCT	142	9.18 ± 1.29	23.53 ± 0.19	-2.74 ± 0.39	70:72	13m	1m	China	Y

All data shown in mean±SD, if not marked otherwise

N: number of participants; COI: report conflict of interest; RCT: randomized controlled trial; m: month; y: year; NA: not mentioned

ALS: axial length shortening

NALS: no axial length shortening

Table S3B. Characteristic of included studies of multifocal soft contact lenses

First author	Year	Study Design	N	Age	Baseline AL (mm)	Baseline SE (D)	Male: Female	Treatment Duration	Cessation Duration	Country	COI
Weng (45)	2022	RCT	MiSight: 34	10.8 ± 1.6	24.5 ± 0.8	-1.91 ± 0.72	16:18	6m	6m	China	Y
Ruiz-Pomeda (46)	2021	RCT	MiSight: 18	13.2 ± 1.28	24.07 ± 0.52	−2.13 ± 0.84	NA	2y	1y	Spain	Y
Cheng (47)	2016	RCT	With +SA: 53	9.7 ± 1.05	24.73 ± 0.71	-2.52 ± 1.10	24:29	1-2y	1.5y	China	N
Anstice (48)	2011	RCT	Dual-Focus: 40	13.4 ± 0.85	24.49 ± 0.72	-2.67 ± 1.09	11:29	10m	10m	New Zealand	Y

All data shown in mean±SD, if not marked otherwise

N: number of participants; COI: report conflict of interest; RCT: randomized controlled trial; m: month; y: year; NA: not mentioned

SA: spherical aberration

Table S3C. Characteristic of included studies of peripheral plus spectacle lenses

First author	Year	Study Design	N	Age	Baseline AL (mm)	Baseline SE (D)	Male: Female	Treatment Duration	Cessation Duration	Country	COI
Lam (49)	2023	RCT	DIMS-SV (Group2): 14	10.21 ± 1.53	25.00 ± 0.8	-2.98 ± 1.13	11:3	3.5y	2.5y	Hong Kong	Y
			SV-DIMS-SV (Group4): 18	10.33 ± 1.71	24.42 ± 0.86	-2.65 ± 1.18	9:9	1.5y	2.5y		
Sankaridrug (50)	2022	RCT	HAL-SV (Group1): 54	11.2 ± 1.6	25.1 ± 0.8	-3.47 ± 1.16	31:23	6m	6m	Vietnam	Y

All data shown in mean±SD, if not marked otherwise

N: number of participants; COI: report conflict of interest; RCT: randomized controlled trial; m: month; y: year

DIMS: Defocus Incorporated Multiple Segments

SV: single-vision spectacles

HAL: Highly aspherical lenslets

Table S4. Detailed quality assessment of included studies using Cochrane risk of bias 2 tool

First Author	Year	Randomization process	Intervention adherence	Missing outcome data	Outcome measurement	Selective reporting	Overall
**Swarbrick HA (37)**	2014	L	H	S	L	L	H
**Wei S (40)**	2017	L	H	S	L	S	H
**Zhu Q (44)**	2023	S	H	S	L	S	H
**Ruiz-Pomeda (46)**	2021	S	H	S	L	L	H
**Cheng (47)**	2016	S	H	S	L	L	H
**Weng (45)**	2022	S	H	S	L	L	H
**Anstice (48)**	2011	L	H	S	L	L	H
**Lam (49)**	2023	L	H	S	L	L	H
**Sankaridrug (50)**	2022	L	H	S	L	L	H

H, high risk of bias; L, low risk of bias; S, some concern of risk of bias.

Table S5. Detailed quality assessment of included studies using Cochrane Risk Of Bias In Non-randomised Studies - of Interventions

First Author	Year	A1	A2	A3	A4	A5	A6	A7	Overall
**Cho P (38)**	2016	Moderate	Low	Low	Moderate	Low	Low	Moderate	Moderate
**Santodomingo-Rubido J (39)**	2016	Critical	Serious	Low	Moderate	Low	Low	Moderate	Critical
**Li H (41)**	2019	Critical	Low	Low	Moderate	Low	Low	Moderate	Critical
**Li Z (42)**	2019	Moderate	Low	Low	Moderate	Low	Low	Moderate	Moderate
**Wang A (43)**	2022	Moderate	Moderate	Low	Moderate	Low	Low	Moderate	Moderate

A1: Bias due to confounding

A2: Bias in selection of participants

A3: Bias in classification of interventions

A4: Bias due to deviations from intended interventions

A5: Bias due to missing data

A6: Bias in measurement of outcomes

A7: Bias in selection of the reported results

References

1. Santodomingo-Rubido J, Villa-Collar C, Gilmartin B, Gutiérrez-Ortega R. Short-term changes in ocular biometry and refraction after discontinuation of long-term orthokeratology. Eye Contact Lens. 2014;40(2):84-90.

2. Lau JK, Wan K, Cheung SW, Vincent SJ, Cho P. Weekly Changes in Axial Length and Choroidal Thickness in Children During and Following Orthokeratology Treatment With Different Compression Factors. Transl Vis Sci Technol. 2019;8(4):9.

3. Polse KA, Brand RJ, Schwalbe JS, Vastine DW, Keener RJ. The Berkeley Orthokeratology Study, Part II: Efficacy and duration. Am J Optom Physiol Opt. 1983;60(3):187-98.

4. Huang Y, Li X, Ding C, Chen Y, Chen H, Bao J. Orthokeratology reshapes eyes to be less prolate and more symmetric. Cont Lens Anterior Eye. 2022;45(4):101532.

5. Polse KA, Brand RJ, Vastine DW, Schwalbe JS. Corneal change accompanying orthokeratology. Plastic or elastic? Results of a randomized controlled clinical trial. Arch Ophthalmol. 1983;101(12):1873-8.

6. Wu R, Stapleton F, Swarbrick HA. Residual corneal flattening after discontinuation of long-term orthokeratology lens wear in asian children. Eye Contact Lens. 2009;35(6):333-7.

7. Yang L, Guo X, Xie P. Observation of orthokeratology discontinuation. Zhonghua Yan Ke Za Zhi. 2015;51(3):178-82.

8. Zhao L, Jing L, Li J, Du X. Changes in corneal densitometry after long-term orthokeratology for myopia and short-term discontinuation. PLoS One. 2022;17(2):e0263121.

9. Guo Y, Liu L, Peng L, Fu J, Guo W, Tang P. Effect of overnight orthokeratology lenses on tear film stability in children. Cont Lens Anterior Eye. 2023;46(1):101592.

10. Nieto-Bona A, González-Mesa A, Nieto-Bona MP, Villa-Collar C, Lorente-Velázquez A. Long-term changes in corneal morphology induced by overnight orthokeratology. Curr Eye Res. 2011;36(10):895-904.

11. Nakamura Y, Hieda O, Yokota I, Teramukai S, Sotozono C, Kinoshita S. Comparison of myopia progression between children wearing three types of orthokeratology lenses and children wearing single-vision spectacles. Jpn J Ophthalmol. 2021;65(5):632-43.

12. Lum E, Golebiowski B, Swarbrick HA. Changes in corneal subbasal nerve morphology and sensitivity during orthokeratology: Recovery of change. Ocul Surf. 2017;15(2):236-41.

13. Chen Z, Zhou J, Xue F, Zhou X, Qu X. Increased Corneal Toricity after Long-Term Orthokeratology Lens Wear. J Ophthalmol. 2018;2018:7106028.

14. Wan K, Lau JK, Cheung SW, Cho P. Refractive and corneal responses of young myopic children to short-term orthokeratology treatment with different compression factors. Cont Lens Anterior Eye. 2020;43(1):65-72.

15. Wan K, Yau HT, Cheung SW, Cho P. Corneal thickness changes in myopic children during and after short-term orthokeratology lens wear. Ophthalmic Physiol Opt. 2021;41(4):757-67.

16. Kang P, Swarbrick H. Discontinuation of long term orthokeratology lens wear and subsequent refractive surgery outcome. Cont Lens Anterior Eye. 2017;40(6):436-9.

17. Gispets J, Yébana P, Lupón N, Cardona G, Pérez-Corral J, Pauné J, et al. Efficacy, predictability and safety of long-term orthokeratology: An 18-year follow-up study. Cont Lens Anterior Eye. 2022;45(1):101530.

18. Cho P, Cheung SW. Retardation of myopia in Orthokeratology (ROMIO) study: a 2-year randomized clinical trial. Invest Ophthalmol Vis Sci. 2012;53(11):7077-85.

19. Soni PS, Nguyen TT, Bonanno JA. Overnight orthokeratology: refractive and corneal recovery after discontinuation of reverse-geometry lenses. Eye Contact Lens. 2004;30(4):254-62; discussion 63-4.

20. Kobayashi Y, Yanai R, Chikamoto N, Chikama T, Ueda K, Nishida T. Reversibility of effects of orthokeratology on visual acuity, refractive error, corneal topography, and contrast sensitivity. Eye Contact Lens. 2008;34(4):224-8.

21. Hiraoka T, Okamoto C, Ishii Y, Okamoto F, Oshika T. Recovery of corneal irregular astigmatism, ocular higher-order aberrations, and contrast sensitivity after discontinuation of overnight orthokeratology. Br J Ophthalmol. 2009;93(2):203-8.

22. Xing XH, Wang XY, Wang SH, Tu J. Influence of the duration of orthokeratology lens cessation on patients' refractive status and corneal endothelial cells. Int Eye Sci. 2023;23(4).

23. Dong J, Liu ZY, Feng YL. The comparative study of orthokeratology lens and frame glasses in adolescent myopia. Chinese Journal of Strabismus & Pediatric Ophthalmology. 2013;21(1).

24. Jin YM, Zhang Y, Li H, Hu BY. Effects of Orthokeratology Lens on Corneal Endothelium and Corneal Thickness. Med J PUMCH. 2011;2(2).

25. Mai ZC, Lin ZX. Study on the Changes of Refraction and Corneal Shape after Stop Wearing Orthokeratology. Mod Diagn Treat. 2016;27(13).

26. Zhou ZX, Xu SS, Yi SP. Clinical effect of orthokeratology for juvenile with myopia astigmatism and its effects on corneaI endotheliaI cells. Int Eye Sci. 2016;16(8).

27. Yang L, Guo X, Xie P. Observation of orthokeratology discontinuation. Chin J Ophthalmol. 2015;0(3).

28. Lu WW, Lian Y, X.L. Y, W.Q. J. Changes and Relationship Analysis of Ocular Parameters After Short-term Discontinuation of Orthokeratology. Journal of Sun Yat-sen University (Medical Sciences). 2019;40(5).

29. Hu J, Geng L, Yang QH, Wamg LQ, Huang YF. Analysis of corneal morphology after one-month discontinuation of orthokeratology treatment. Acad J Chin PLA Med Sch. 2019;40(9).

30. Sibug ME, Datiles MB, 3rd, Kashima K, McCain L, Kracher G. Specular microscopy studies on the corneal endothelium after cessation of contact lens wear. Cornea. 1991;10(5):395-401.

31. Lei Y, Zheng X, Hou J, Xu B, Mu G. Effects of long-term soft contact lens wear on the corneal thickness and corneal epithelial thickness of myopic subjects. Mol Med Rep. 2015;11(3):2020-6.

32. Sankaridurg P. Contact lenses to slow progression of myopia. Clin Exp Optom. 2017;100(5):432-7.

33. Zhu Q, Liu Y, Tighe S, Zhu Y, Su X, Lu F, et al. Retardation of Myopia Progression by Multifocal Soft Contact Lenses. Int J Med Sci. 2019;16(2):198-202.

34. Morgan PB, Sulley AL. Challenges to the new soft contact lens wearer and strategies for clinical management. Cont Lens Anterior Eye. 2023;46(3):101827.

35. Ruiz-Pomeda A, Pérez-Sánchez B, Prieto-Garrido FL, Gutiérrez-Ortega R, Villa-Collar C. MiSight Assessment Study Spain: Adverse Events, Tear Film Osmolarity, and Discontinuations. Eye Contact Lens. 2018;44 Suppl 2:S180-s6.

36. Berntsen DA, Mutti DO, Zadnik K. Study of Theories about Myopia Progression (STAMP) design and baseline data. Optom Vis Sci. 2010;87(11):823-32.

37. Swarbrick HA, Alharbi A, Watt K, Lum E, Kang P. Myopia control during orthokeratology lens wear in children using a novel study design. Ophthalmology. 2015;122(3):620-30.

38. Cho P, Cheung SW. Discontinuation of orthokeratology on eyeball elongation (DOEE). Cont Lens Anterior Eye. 2017;40(2):82-7.

39. Santodomingo-Rubido J, Villa-Collar C, Gilmartin B, Gutiérrez-Ortega R, Sugimoto K. Long-term Efficacy of Orthokeratology Contact Lens Wear in Controlling the Progression of Childhood Myopia. Curr Eye Res. 2017;42(5):713-20.

40. Wei S, Li SM, An W, Du J, Liang X, Sun Y, et al. Myopia progression after cessation of low-dose atropine eyedrops treatment: A two-year randomized, double-masked, placebo-controlled, cross-over trial. Acta Ophthalmol. 2023;101(2):e177-e84.

41. Li H, Ouyang J. The effect of long-term wearing of keratoplasty lenses and stopping wearing on corneal thickness. Proceeding of Clinical Medicine. 2019;28(7):496-9.

42. Li Z, Hu Y, Cui D, Long W, He M, Yang X. Change in subfoveal choroidal thickness secondary to orthokeratology and its cessation: a predictor for the change in axial length. Acta Ophthalmol. 2019;97(3):e454-e9.

43. Wang A, Yang C, Shen L, Wang J, Zhang Z, Yang W. Axial length shortening after orthokeratology and its relationship with myopic control. BMC Ophthalmol. 2022;22(1):243.

44. Zhu Q, Yin J, Li X, Hu M, Xue L, Zhang J, et al. Effects of Long-Term Wear and Discontinuation of Orthokeratology Lenses on the Eyeball Parameters in Children with Myopia. Int J Med Sci. 2023;20(1):50-6.

45. Weng R, Lan W, Bakaraju R, Conrad F, Naduvilath T, Yang ZK, et al. Efficacy of contact lenses for myopia control: Insights from a randomised, contralateral study design. Ophthalmic Physiol Opt. 2022;42(6):1253-63.

46. Ruiz-Pomeda A, Prieto-Garrido FL, Hernández Verdejo JL, Villa-Collar C. Rebound Effect in the Misight Assessment Study Spain (Mass). Curr Eye Res. 2021;46(8):1223-6.

47. Cheng X, Xu J, Chehab K, Exford J, Brennan N. Soft Contact Lenses with Positive Spherical Aberration for Myopia Control. Optom Vis Sci. 2016;93(4):353-66.

48. Anstice NS, Phillips JR. Effect of dual-focus soft contact lens wear on axial myopia progression in children. Ophthalmology. 2011;118(6):1152-61.

49. Lam CSY, Tang WC, Zhang HY, Lee PH, Tse DYY, Qi H, et al. Long-term myopia control effect and safety in children wearing DIMS spectacle lenses for 6 years. Sci Rep. 2023;13(1):5475.

50. Sankaridurg P, Weng R, Tran H, Spiegel DP, Drobe B, Ha T, et al. Spectacle Lenses With Highly Aspherical Lenslets for Slowing Myopia: A Randomized, Double-Blind, Cross-Over Clinical Trial: Parts of these data were presented as a poster at the Annual Research in Vision and Ophthalmology meeting, 2022. Am J Ophthalmol. 2023;247:18-24.
